# Evaluation of the Penetration Depth of Four Different Root Canal Sealers into Dentinal Tubules: A Scanning Electron Microscopic Study

**DOI:** 10.7759/cureus.102511

**Published:** 2026-01-28

**Authors:** Swapna Sannapureddy, Nusrath Parveen, Suneel Kumar Chinni, Lavanya Anumula, Niharika Mungara

**Affiliations:** 1 Conservative Dentistry and Endodontics, Narayana Dental College and Hospital, Nellore, IND; 2 Endodontics, SS Dental Care Multi-Specialty Dental Clinic and Root Canal Centre, Sullurpeta, IND

**Keywords:** ah plus sealer, apexit plus, mta fillapex, scanning electron microscope (sem), tubular penetration

## Abstract

Aim

This study was designed to establish the average depth of penetration of four different root canal sealers into the dentinal tubules at the cervical, middle, and apical third levels.

Methodology

Forty-eight mandibular premolars were decoronated, and cleaning and shaping were done in a crown-down manner using ProTaper Gold (Densply Maillefer, Ballaigues, Switzerland) rotary instruments. To simulate smear layer removal in a clinical setting, all canals were then irrigated with 3 ml of 17% ethylenediaminetetraacetic acid (EDTA), i.e., SmearClear (Sybron Endo, Glendora, CA, USA) for one minute, followed by 3 ml of 3% sodium hypochlorite. Canal irrigation was performed with 5 ml of saline to get rid of excess irrigating solution. All the samples were randomised into four groups based on the sealer by a person not related to the study to ensure anonymity, as follows: Group A: Zinc oxide eugenol sealer (ZOE); Group B: AH Plus sealer (Densply Maillefer); Group C: Apexit Plus sealer (Ivoclaar vivadent, Gurugram, India); Group D: mineral trioxide aggregate (MTA) Fillapex sealer (Angelus, Torrance, CA, USA). Obturation of the samples was done using the lateral compaction, and samples were then sectioned in the bucco-lingual mode using a disc under copious irrigation using distilled water. Samples were dehydrated and gold sputtered for scanning electron microscopy (SEM) evaluation. Data was pooled and interpreted using the Mann-Whitney U test and Kruskal-Wallis test.

Results

Analysis showed a statistically significant difference with a p-value of <0.05 between the coronal third, middle third, and apical third in all the groups. In pairwise comparison, the mean depth of penetration of MTA Fillapex was much higher than that of Apexit Plus, AH Plus, and ZOE sealer at the coronal, middle third, and apical third levels.

Conclusion

Within the limitations of this in vitro SEM study, MTA Fillapex demonstrated superior dentinal tubule penetration compared with AH Plus, Apexit, and zinc oxide eugenol-based sealer.

## Introduction

The ultimate aim of endodontic procedures is the elimination of intracanal microorganisms, followed by the prevention of reinfection by providing a proper apical and coronal seal using modern obturation materials [1]. Successful obturation is achieved using materials that are biologically acceptable as well as able to provide dimensional stability within the root canal system [2]. Insufficient filling material adaptation within the complex internal anatomy of the canal space, especially within the apical part, has been identified as a prominent reason for endodontic treatment failure [3]. Gutta-percha combined with a root sealer is the most commonly utilized obturating material in dentistry. The role of these sealers is vital within the process of obturation, as they help in compensating for differences in dimensions between the sides of the canal walls and gutta-percha, thus improving the obturation quality [4].

A critical attribute of an optimal root canal sealer is its ability to achieve an optimal seal through penetration into dentine tubules, thereby promoting adaptation of the material to the canal walls [5]. Classification of root canal sealers based on their composition is done as follows: zinc oxide and eugenol root canal sealers, calcium hydroxide root canal sealers, resin-based root canal sealers, glass ionomer root canal sealers, mineral trioxide aggregate root canal sealers, and bioceramic root canal sealers. Of late, zinc oxide and eugenol root canal sealers act as the standard in assessing newer types of root canal sealers. But their use has been hampered by drawbacks such as leakage and an adhesion and adaptation ability that reduces over time [6].

The formulation of Apexit Plus, a calcium hydroxide-based root canal sealer, includes salicylate, which influences its handling properties in clinical practice [7]. Epoxy resin-based sealers have been widely used in endodontic practices, with AH 26 and its modified version, AH Plus, being some of the most widely studied materials. Due to its desirable properties of sealing and dimensional stability, AH Plus is frequently used as an obturating material in comparative studies with resin-based sealers [8]. MTA Fillapex is an MTA-based sealer, developed to provide the biological properties of MTA with improved handling properties such as satisfactory flow, radiopacity, longer set times, and reduced solubility. Its desirable physical properties, along with good biocompatibility and bioactivity, have encouraged its use in clinical settings [9, 10]. Hence, this research aims to compare the penetration of dentinal tubules of four different root sealers at the coronal, middle, and apical thirds using scanning electron microscopy (SEM) in relation to their depths of penetration.

## Materials and methods

The institutional ethical board of the Narayana Dental College and Hospital approved this in vitro study protocol under reference number D168401010.

Sample size calculation

The sample size was calculated using G*Power software (Ver. 3.1.9.7, Heinrich-Heine-Universität, Düsseldorf, Germany). Based on an alpha error probability of 0.05 and a power of 80% (β=0.20), a total of 48 samples (12 per group) was determined to be sufficient to detect a large effect size approximately equal to 0.58 for differences between the four experimental groups and the three root levels (coronal, middle, and apical).

Inclusion and exclusion criteria

Human mandibular premolars with a standardized length of 20 mm extracted for orthodontic purposes from the patients in the age group of 18-25 years that were fully developed, had closed apices, were caries-free, and were devoid of cracks and resorption were included. Multirooted teeth, endodontically treated teeth, and developmental anomalies were excluded.

Procedure

Based on the G Power analysis, 48 freshly extracted human mandibular premolars for orthodontic reasons were selected, and the crowns of each sample were sliced at the cemento-enamel junction using a water-cooled carborundum disc to a standardized root length of 12 mm. The patency to the root canal was established, and pulp residues were removed using barbed broaches. Canal length was measured using a #15 stainless steel K-file (25 mm, Densply Maillefer, Ballaigues, Switzerland), from which 1 mm was subtracted when its tip came out at the apical foramen.

ProTaper Gold nickel-titanium rotary instruments (Dentsply Maillefer) were used in the crown-down method of chemomechanical preparation. Shaping was completed with an F3 file (size 30, 0.06 taper) at the specified working length. Five mL of 3% sodium hypochlorite solution (Prime Dental Products) was used to irrigate the canals between each instrument. Throughout the process, apical patency was preserved by passing a #15 K-file past the apical constriction.

Following instrumentation, smear layer removal was performed by irrigating each canal with 3 mL of 17% ethylenediaminetetraacetic acid (EDTA) (SmearClear; Sybron Endo, Glendora, CA, USA) for one minute. Then, 3 mL of 3% sodium hypochlorite was used for irrigation, and a final rinse was carried out using 5 mL of saline solution. Then the sterile absorbent paper points were used to dry the canals. Depending on the type of root canal sealer applied, the specimens were randomly divided into four experimental groups using computer-generated randomization (n=12 per group): Group A: zinc oxide eugenol-based sealer; Group B: AH Plus sealer (Dentsply); Group C: Apexit Plus sealer (Ivoclar Vivadent, Gurugram, India); and Group D: MTA Fillapex sealer (Angelus, Torrance, CA, USA).

**Figure 1:**
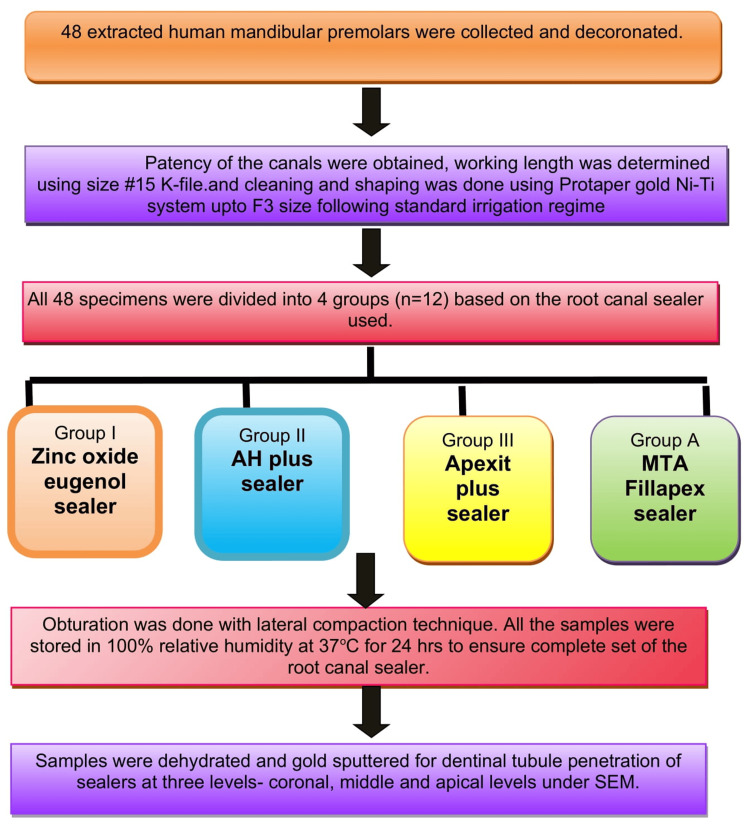
The methodology flowchart MTA: Mineral trioxide aggregate; SEM: scanning electron microscopy.

The amount of sealer placed within the root canal was standardized by adopting a controlled placement technique by employing a lentulo spiral inserted 1 mm short of working length mounted to a low-speed handpiece rotated at 300 rpm. Obturation was performed using a master cone of 6% gutta-percha along with accessory cones to compensate for voids, employing the lateral compaction technique. The quality of obturation was verified using radiovisiography, and the access cavities were sealed with an intermediate restorative material (Cavit G; Solventum, St. Paul, MN, USA). All specimens were kept for 24 hours at 37°C with 100% relative humidity to allow the sealers to fully set in a humidity chamber. The detailed methodology flow chart was presented in Figure 1. 

Scanning electron microscope evaluation

**Figure 2:**
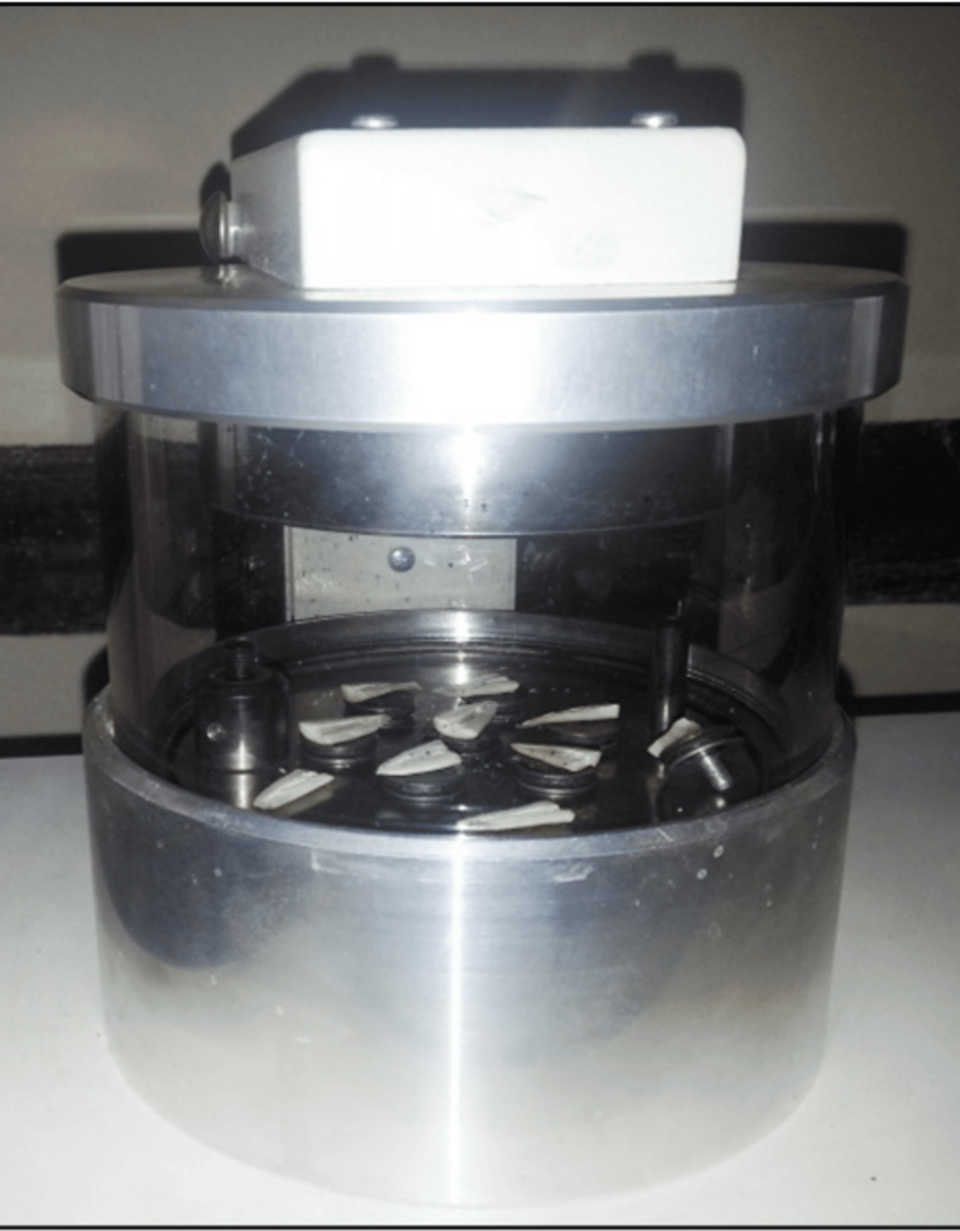
Gold sputtering for scanning electron microscopic (SEM) evaluation

A safe-sided cutting disc (Jinguang double-sided dental discs, Wuhan Jinguang Medical Technology Co., Ltd., Wuhan, China) was used to segment the samples in the bucco-lingual direction under copious irrigation with distilled water. A scanning electron microscope was used to examine the half that retained the obturation material. The tooth halves were immersed in EDTA solution for 10 minutes, followed by immersion in 3% NaOCl solution for 10 minutes, and then cleaned thoroughly with distilled water. Afterward, the samples were gold-sputtered and kept in an ultra-high vacuum chamber (UHV) programmed at 15 kV for SEM evaluation, as shown in Figure 2. 

Evaluation of sealer penetration

Sealer penetration into dentinal tubules was evaluated across three distinct regions: the coronal, middle, and apical thirds. The sealer dentin junction was taken as a reference point up to the maximum penetration depth. Images were captured and measured using ImageJ software (National Institutes of Health (NIH), Bethesda, MD, USA) at 500X-2500X magnification. For each experimental sample, lines were drawn from the canal wall to the highest and minimum locations of visible sealer penetration into the dentinal tubules, calibrated using the embedded scale bar to convert pixel values to micrometers. The study group allocations were concealed from both the statistician and the operator performing SEM evaluation. Each SEM image was analyzed by two blinded operators determining dentinal tubules filled with sealer, and the depth of penetration identified the dentinal tubules filled with sealer. To ensure consistency, the inter-observer reliability was assessed using Cohen's kappa statistics; the kappa value is 0.72, suggesting excellent reliability.

Statistical analysis

Data analysis was carried out using the Statistical Package for Social Sciences (SPSS version 23; IBM Corp., Armonk, NY, USA). The Shapiro-Wilk tests were used to determine whether the continuous data were normal and are presented in Table 1. The mean and standard deviation (SD) were used to characterize continuous data. The significance level was set at p<0.05. Although the data demonstrated normal distribution, the Kruskal-Wallis test was used due to the small sample size per group and inherent biological variability of dentinal tubule penetration measurements, with the Mann-Whitney U test applied for pairwise comparisons.

**Table 1:** Normality of the data was evaluated using the Shapiro-Wilk test. ZOE: Zinc oxide eugenol; MTA: mineral trioxide aggregate; df: degrees of freedom.

Sealer	Root level	W statistic	df	p value	Distribution
ZOE	Coronal	0.952	12	0.672	Normal
ZOE	Middle	0.970	12	0.910	Normal
ZOE	Apical	0.925	12	0.326	Normal
AH Plus	Coronal	0.914	12	0.241	Normal
AH Plus	Middle	0.929	12	0.374	Normal
AH Plus	Apical	0.976	12	0.961	Normal
Apexit Plus	Coronal	0.931	12	0.390	Normal
Apexit Plus	Middle	0.939	12	0.484	Normal
Apexit Plus	Apical	0.971	12	0.917	Normal
MTA Fillapex	Coronal	0.962	12	0.809	Normal
MTA Fillapex	Middle	0.952	12	0.661	Normal
MTA Fillapex	Apical	0.970	12	0.914	Normal

## Results

The overall penetration of sealers at the coronal, middle, and apical thirds was presented in Figure 2.

**Figure 3:**
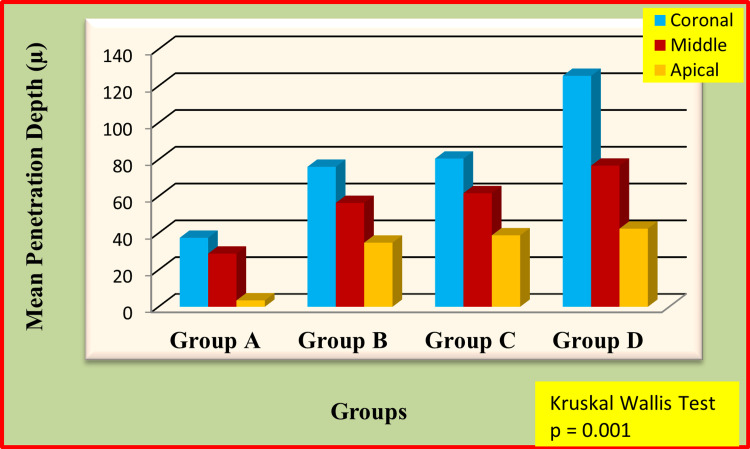
Overall comparison of penetration of different sealers at coronal, middle and apical portions of the teeth X-axis represents groups, Y-axis represents mean penetration depth of the sealers

The sealer penetration was significantly higher at the coronal third of the root canal when compared to the middle and apical portions of the teeth in all the groups. Pairwise comparison of penetration of four different sealers at coronal portions of the teeth was tabulated in Table 2.

**Table 2:** Pairwise comparison of penetration of four different sealers at coronal portions of the teeth Mann-Whitney test, P<0.008 (significant), P>0.008 (not significant) (Bonferroni adjusted). * indicates significant difference.

Groups	Mean penetration depth (µ)	p value
Group A vs Group B	37.53 vs 76.09	p = 0.001*
Group A vs Group C	37.53 vs 80.56	p = 0.001*
Group A vs Group D	37.53 vs 125.55	p = 0.001*
Group B vs Group C	76.09 vs 80.56	p = 0.184
Group B vs Group D	76.09 vs 125.55	p = 0.001*
Group C vs Group D	80.56 vs 125.55	p = 0.001*

The zinc oxide eugenol sealer showed significantly less sealer penetration when compared to the other three sealers in the coronal, middle, and apical thirds. The multiple comparison of sealer penetration at the middle third level was shown in Table 3.

**Table 3:** Pair wise comparison of penetration of four different sealers at middle portions of the teeth Mann-Whitney test, P<0.008 (Significant), P>0.008 (Not significant) (Bonferroni adjusted), *shows significant difference.

Groups	Mean penetration depth (µ)	p value
Group A vs Group B	28.89 vs 56.45	0.001*
Group A vs Group C	28.89 vs 61.75	0.001*
Group A vs Group D	28.89 vs 76.72	0.001*
Group B vs Group C	56.45 vs 61.75	0.003*
Group B vs Group D	56.45vs76.72	0.001*
Group C vs Group D	61.75vs76.72	0.001*

MTA Fillapex showed significantly more penetration than AH Plus and Apexit Plus at all the levels. There is no significant difference between AH Plus and Apexit Plus. The apical third comparison for four different sealers was tabulated in Table 4.

**Table 4:** Pair wise comparison of penetration of four different sealers at apical portions of the teeth Mann-Whitney test, P<0.008 (Significant), P>0.008 (Not significant).

Groups	Mean penetration depth (µ)	p value
Group A vs Group B	3.49 vs 34.85	p = 0.001*
Group A vs Group C	3.49 vs 38.85	p = 0.001*
Group A vs Group D	3.49 vs 42.47	p = 0.001*
Group B vs Group C	34.85 vs 38.85	p = 0.028
Group B vs Group D	34.85 vs 42.47	p = 0.009
Group C vs Group D	38.85 vs 42.47	p = 0.106

## Discussion

Successful endodontic therapy depends not only on the cleaning and shaping of the root canal system but also on the ability of obturation materials to achieve a three-dimensional seal. Penetration of root canal sealers into dentinal tubules plays a crucial role in enhancing mechanical interlocking, reducing microleakage, entombing residual microorganisms, and improving long-term sealing ability [11,12]. Therefore, the present SEM study compared the penetration depth of four commonly used root canal sealers - zinc oxide eugenol-based sealer, AH Plus, Apexit, and MTA Fillapex - into dentinal tubules.

The findings demonstrated a consistent pattern of penetration depth, with MTA Fillapex showing the highest penetration, followed by Apexit Plus and AH Plus, while ZOE exhibited the least penetration (see Appendices). This order of penetration corroborates recent studies indicating that sealers with higher flowability, finer particle size, and lower film thickness demonstrate enhanced intratubular penetration, particularly in the coronal and middle thirds. These results are in accordance with Nikhil et al. [13] and Kuçi et al. [14], where MTA Fillapex showed significantly better penetration than AH Plus. Owing to the better flow and less viscous nature of MTA Fillapex. Zhou et al. [15] and Silva et al. [16] observed that the flow of MTA Fillapex is greater than that of AH Plus sealer. The difference in composition and smaller particle size of the sealer contributed to better flow in MTA Fillapex. The reason for this result could be because of better flow of MTA Fillapex.

The recent literature by Tosun et al. concluded that MTA Fillapex and BioRoot Flow exhibited superior performance compared to AH Plus [17]. A study by Karataşlioğlu and Tosun stated that MTA Fillapex exhibited a significantly greater penetration area than AH Plus when ultrasonic and XP-Endo Finisher activation was applied [18].

The resin sealers, such as AH Plus, continue to be considered a gold standard due to their favorable flow characteristics, low solubility, dimensional stability, and consistent adaptation to dentinal walls [19]. The results of the present study were contrary to the literature by Gülmez et al. in which AH Plus showed more penetration depth and area in the coronal section than MTA Fillapex [20].

Apexit Plus demonstrated intermediate penetration values in the present study. Calcium hydroxide-based sealers benefit from relatively low viscosity, facilitating dentinal tubule entry; however, their higher solubility and potential for long‑term dissolution may compromise sealing durability. Recent literature highlights that the penetration performance of Apexit Plus is strongly influenced by smear layer removal protocols and obturation techniques [21].

MTA Fillapex exhibited the greatest dentinal tubule penetration, particularly in the coronal and middle thirds. Its salicylate resin matrix, nanoparticulate MTA components, and superior flow characteristics have been proposed as contributing factors. While deeper penetration may enhance mechanical interlocking and bacterial entombment, several studies caution that penetration depth alone does not necessarily predict long‑term sealing efficacy, emphasizing the importance of evaluating solubility, dimensional stability, and bond strength [22].

ZOE sealers consistently demonstrated the lowest penetration depth. Their relatively coarse particle size, increased film thickness, and limited flow reduce their ability to infiltrate dentinal tubules. Although ZOE sealers have a long history of clinical use, their inferior penetration and higher solubility in moist environments may limit their capacity to entomb residual intratubular bacteria when compared with resin- and calcium silicate-based sealers [23].

Across all groups, sealer penetration decreased from coronal to apical thirds, reflecting the reduced density and diameter of dentinal tubules toward the apex, increased dentin sclerosis, and limited effectiveness of irrigants in the apical region. These findings align with established dentin morphology and underscore the clinical challenge of achieving effective apical sealing. Clinically, deeper penetration of sealers into dentinal tubules may contribute to improved sealing ability and long-term success of root canal treatment.

The limitations of this study are the small sample size, the in vitro design not stimulating the clinical functional loading, the lack of ultrasonic irrigation activation, and the usage of a single obturation technique. Advanced assessment methods like confocal scanning electron microscopy were not included; no volumetric analysis for the amount of the sealer was done, and the penetration depth alone should not be considered the sole determinant of sealer performance, as other factors such as dimensional stability, solubility, biocompatibility, and antimicrobial properties also play critical roles.

The clinical implications are that greater penetration of root canal sealers into dentinal tubules enhances interfacial sealing, reduces microleakage, and contributes to long-term resistance against bacterial reinfection, further avoiding endodontic retreatments in the future.

## Conclusions

Within the limitations, the current study concluded that the penetration of the sealers was influenced by the type of the sealer, composition, and flow. The mean penetration depth of sealers in the coronal third was more than that of the middle third and apical third. Overall, MTA Fillapex showed the highest penetration depth into the dentinal tubules when compared to the other three sealers. These findings warrant further investigation into the correlation between penetration depth and fluid-tight seal for these materials.

## Appendices

**Table 5:** Overall comparison of penetration of four different sealers at coronal, middle and apical portions of the teeth Kruskal-wallis test, P<0.05 (significant), P>0.05 (not significant).

Groups	Frequency	Mean±SD (µ)	Statistical test and p value
Coronal			Kruskal-Wallis Test. p=0.001
Group A	12	37.53±4.66	
Group B	12	76.09±8.31	
Group C	12	80.56±5.87	
Group D	12	125.55±14.87	
Middle			Kruskal-Wallis Test, p=0.001
Group A	12	28.89±3.61	
Group B	12	56.45±4.15	
Group C	12	61.75±3.65	
Group D	12	76.72±10.73	
Apical			Kruskal-Wallis Test, p=0.001
Group A	12	3.49±0.94	
Group B	12	34.85±4.26	
Group C	12	38.85±3.39	
Group D	12	42.47±7.018	
